# Geographic Difference of Mortality of Creutzfeldt-Jakob Disease in Japan

**DOI:** 10.2188/jea.17.19

**Published:** 2006-12-29

**Authors:** Yosikazu Nakamura, Makoto Watanabe, Kiwamu Nagoshi, Masahito Yamada, Hidehiro Mizusawa

**Affiliations:** 1 Department of Public Health, Jichi Medical University.; 2 Department of Neurology and Neurobiology of Aging, Kanazawa University Graduate School of Medical Science.; 3 Department of Neurology and Neurological Science, Tokyo Medical and Dental University.

**Keywords:** Creutzfeldt-Jakob Syndrome, Mortality, Geographic Locations, Cluster Analysis, Japan

## Abstract

**BACKGROUND:**

The geographic mortality difference of Creutzfeldt-Jakob disease is still unclear in Japan.

**METHODS:**

Using vital statistics of Japan for 6 year period between 1999 and 2004 officially published by the government, we observed the mortality from Creutzfeldt-Jakob disease (ICD-10th: A81.0 and A81.8) by prefecture. Standardized mortality ratios were calculated for the 47 prefectures.

**RESULTS:**

For the observed 6 years, a total of 792 deaths from Creutzfeldt-Jakob disease were observed whole in Japan. Two prefectures, Akita and Yamanashi, presented significantly high standardized mortality ratios. In addition, Tochigi, Kochi, and Nagasaki showed standardized mortality rates higher than 1.5 without significance. No prefecture had significantly low standardized mortality ratios.

**CONCLUSION:**

Some prefectures with high mortality rate from Creutzfeldt-Jakob disease existed in Japan. Some of them had high incidence rate in a survey conducted in 1996 as well.

To clarify whether there were patients with variant Creutzfeldt-Jakob disease (CJD) in Japan, a nationwide epidemiologic survey was conducted in 1996,^1^ when the relationship between the variant CJD and bovine spongiform encephalopathy was reported from the United Kingdom. In the survey, some prefectures showed high incidence rate, which means that geographical difference of the frequency of CJD exists in Japan. Although Japanese government has continued a surveillance of prion diseases since then because the problem of iatrogenic CJD manifests itself in the survey,^2^ the geographical difference of the disease has not been discussed. On the other hand, vital statistics in Japan started to provide the number of deaths from CJD by prefecture in 1999.

We consider it important to clarify the geographic difference of CJD in Japan using the 6-year vital statistics (1999-2004) in Japan.

## METHODS

We used vital statistics of Japan officially published by the government, and observed Creutzfeldt-Jakob disease (ICD-10th: A81.0 [Creutzfeldt-Jakob disease] and A81.8 [Other atypical virus infections of central nervous system]). Six-year data beginning in 1999, when the government started to present a table of the number of deaths from CJD by prefecture, and ending in 2004, which is the most recent year when such data are available, were used. Actually, the fatal number by prefecture is published as a combined figure of A81.0 and A81.8 with a code for infectious diseases "In417" in 1999-2002, and "In512" in 2003 and 2004. In Japan, data on death certificates issued by physicians are finally gathered in the Statistics and Information Department, Minister's Secretariat, Ministry of Health, Labour and Welfare of the Japanese government through public health centers and prefectural governments, and are coded in the Statistics and Information Department according the International Classification of Diseases issued by the World Health Organization. First we obtained the yearly numbers of deaths from CJD (Table 1) and the number of deaths due to CJD by prefecture for the 6 years. Then we got the number of deaths due to the disease by age and sex from the Department, which has been prepared for public. Using the population of Census 2000 by age and sex, age- and sex-specific mortality rate of CJD in Japan was calculated, as shown in Table 2. The standard mortality ratio (SMR) and its 95% confidence interval (CI) were calculated by prefecture with the age- and sex-specific mortality rate. The 95% CI were calculated using a table Schoenberg BS prepared.^3^

**Table 1.** The yearly number of patients with Creutzfeldt-Jakob disease by sex and disease, Japan, 1999-2004.

Calendar year	No. of deaths						Crude mortality rate^*^		
	
	Creutzfeldt-Jakob disease (ICD-10th: A81.0)			Other atypical virus infections of central nerveous system (ICD-10th: A81.8)			Creutzfeldt-Jakob disease (ICD-10th: A81.0 + A81.8)		
		
	Male	Female	Total	Male	Female	Total	Male	Female	Total
1999	51	61	112		3	3	0.83	1.00	0.92
2000	44	68	112		1	1	0.72	1.08	0.90
2001	61	61	122	1		1	1.01	0.95	0.97
2002	53	80	133			1	0.88	1.24	1.06
2003	70	71	141	1	1	1	1.14	1.12	1.13
2004	65	96	161	3	1	4	1.10	1.49	1.31

Total	344	437	781	5	6	11	0.95	1.15	1.05

*: /million population/year

**Table 2.** The number of deaths and mortality rate of Creutzfeldt-Jakob disease (ICD-10th: A81.0 + A81.8) by age and sex, Japan, 1999-2004.

	No. of deaths (1999-2004) (a)		Population^*^ (b)		Mortality rate^**^ (a/b/6)	
		
Age (year)	Male	Female	Male	Female	Male	Female
0-4			3,022,521	2,881,577		
5-9			3,083,431	2,938,358		
10-14			3,353,150	3,193,462		
15-19			3,833,984	3,654,181		
20-24	1		4,307,242	4,114,218	0.04	
25-29		2	4,965,277	4,825,032		0.07
30-34	4	1	4,436,818	4,339,792	0.15	0.04
35-39			4,096,286	4,018,579		
40-44	5	5	3,924,171	3,876,048	0.21	0.21
45-49	8	8	4,467,772	4,448,236	0.30	0.30
50-54	13	18	5,210,038	5,231,952	0.42	0.57
55-59	42	44	4,290,239	4,443,933	1.63	1.65
60-64	54	66	3,749,528	3,986,305	2.40	2.76
65-69	73	83	3,357,281	3,748,658	3.62	3.69
70-74	72	90	2,670,270	3,230,306	4.49	4.64
75-79	48	77	1,625,822	2,524,778	4.92	5.08
80-84	20	36	915,268	1,699,421	3.64	3.53
85-89	8	10	477,083	1,055,240	2.79	1.58
90+	1	3	176,392	524,633	0.94	0.95

Total	349	443	62,110,764	64,815,079	0.94	1.14

* : Census 2000

** : /million population/year

## RESULTS

For the observed 6 years, a total of 792 deaths from CJD were observed whole in Japan. As shown in Table 1, the crude mortality rate of CJD in Japan increased gradually.

Table 3 shows the observed and expected numbers of deaths from CJD, SMRs and their 95% CIs for each prefecture. Figure shows the geographic distribution of the SMRs by prefecture. Two prefectures, Akita and Yamanashi, presented significantly high SMRs. In addition, Tochigi, Kochi and Nagasaki showed SMRs higher than 1.5 without significance. No prefecture had significantly low SMRs.

**Table 3.** The observed and expected numbers of deaths, and standardized mortality ratio of Creutzfeldt-Jakob disease (ICD-10th: A81.0 + A81.8) by prefecture, Japan, 1999-2004.

	No. of deaths		

	observed	expected	standardized mortality ratio (95% confidence interval)
total	792	792	
Hokkaido	37	36.9	1.00 (0.70 - 1.38)
Aomori	6	10.0	0.60 (0.22 - 1.30)
Iwate	11	10.3	1.07 (0.53 - 1.91)
Miyagi	18	14.5	1.24 (0.74 - 1.96)
Akita	20	9.3	2.14 (1.31 - 3.29)
Yamagata	7	9.4	0.74 (0.30 - 1.53)
Fukushima	14	14.6	0.96 (0.52 - 1.61)
Ibaraki	13	18.0	0.72 (0.38 - 1.23)
Tochigi	20	12.3	1.62 (0.99 - 2.49)
Gunma	13	13.0	1.00 (0.53 - 1.71)
Saitama	38	36.6	1.04 (0.73 - 1.42)
Chiba	31	33.0	0.94 (0.64 - 1.33)
Tokyo	80	71.0	1.12 (0.89 - 1.39)
Kanagawa	47	46.1	1.02 (0.75 - 1.35)
Niigata	9	17.7	0.51 (0.23 - 0.97)
Toyama	8	7.9	1.01 (0.43 - 1.98)
Ishikawa	7	7.6	0.92 (0.37 - 1.89)
Fukui	4	5.7	0.70 (0.19 - 1.80)
Yamanashi	12	5.9	2.03 (1.05 - 3.56)
Nagano	11	15.9	0.69 (0.35 - 1.24)
Gifu	15	13.6	1.10 (0.61 - 1.81)
Shizuoka	30	24.0	1.25 (0.84 - 1.79)
Aichi	44	39.2	1.12 (0.81 - 1.50)
Mie	9	12.3	0.73 (0.33 - 1.39)
Shiga	8	7.7	1.03 (0.44 - 2.03)
Kyoto	12	16.4	0.73 (0.38 - 1.28)
Osaka	52	51.2	1.01 (0.76 - 1.33)
Hyogo	33	34.2	0.96 (0.66 - 1.35)
Nara	12	8.8	1.36 (0.70 - 2.37)
Wakayama	5	7.7	0.65 (0.21 - 1.51)
Tottori	1	4.4	0.23 (0.01 - 1.25)
Shimane	9	6.0	1.49 (0.68 - 2.83)
Okayama	9	13.3	0.67 (0.31 - 1.28)
Hiroshima	14	18.6	0.75 (0.41 - 1.26)
Yamaguchi	16	11.4	1.40 (0.80 - 2.26)
Tokushima	1	6.0	0.17 (0.00 - 0.93)
Kagawa	5	7.2	0.69 (0.22 - 1.62)
Ehime	6	10.8	0.56 (0.20 - 1.21)
Kochi	11	6.3	1.75 (0.88 - 3.14)
Fukuoka	33	30.8	1.07 (0.73 - 1.49)
Saga	5	6.0	0.84 (0.27 - 1.95)
Nagasaki	16	10.6	1.51 (0.86 - 2.44)
Kumamoto	14	13.1	1.07 (0.58 - 1.80)
Oita	6	8.9	0.67 (0.25 - 1.46)
Miyazaki	3	8.1	0.37 (0.08 - 1.07)
Kagoshima	13	13.1	0.99 (0.53 - 1.69)
Okinawa	4	6.5	0.62 (0.17 - 1.58)

## DISCUSSION

Until now geographic clustering of CJD has been reported from several countries, such as in United Kingdom,4 France,^5^ Italy,^6^ Finland,^7^ the United States,^8^ and Canada.^9^ In Japan, the geographic clustering of CJD has been reported as well.^10^^-^^13^ Some of these clustering might occur because of the familial CJD cases,^14^^,^^15^ but it is important to reveal whether the disease cluster or not as an infectious disease.

The current study shows the similar geographic difference of the result of nationwide incidence survey in 1996 as well as the increasing mortality rate from CJD.^1^ Considering the natural history of CJD, which is that the case-fatality rate during one year from the onset is more than 90%,^16^^,^^17^ the fatal cases observed in the current study differ from those observed in the nationwide survey. Therefore, CJD may be prevalent in the prefecture where high SMRs were observed in the 2 studies.

**Figure 1.**
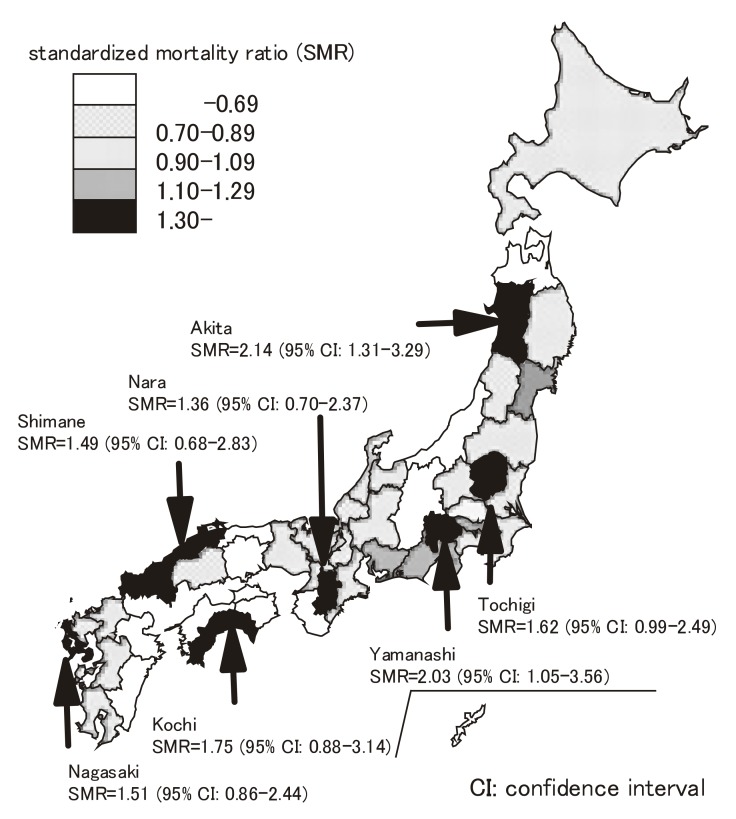
Geographic distribution of standardized mortality ratios of Creutzfeldt-Jakob disease (ICD-10th: A81.0+A81.8), Japan, 1999-2004.

Case ascertainment is the most considerable issue when clustering of diseases is observed.^14^ In Japan, the number of neurologists is so large that the probability of the case finding artifact might be small with the specific clinical findings of the disease.^1^ A total of 115 deaths were reported to the vital statistics as shown in Table 1 in 1999, when the surveillance system of the government newly recognized 84 cases with CJD. In 2000, 113 deaths was compared to the 100 incident cases of the disease followed by 123 death cases and 113 incident cases in 2001, and 134 death cases and 92 incident cases in 2002 (unpublished data). After 2002, the gap of the numbers between death cases and incident cases became large because the surveillance system is now gathering data of the incident cases in these calendar years. As shown above, the two figures, the numbers of deaths and incident cases, were so close that we believe the validity of diagnosis and covering rate of the patients on the vital statistics are not complete but satisfactory to discuss the issue of this study. In addition, death certificates were efficient sources for ascertainment of CJD in the US.^18^

Akita and Tochigi showed high incidence rate in the study of 1996.^1^ The previous study observed the patients with CJD whose onset was from 1985 through 1996 by mail survey to 3965 departments of neurology, psychiatry, and neuropathology of all hospitals with 100 or more beds in Japan. Of these departments, 2899 (73.1%) reported 821 patients. Standardized morbidity ratios were significantly high in three prefectures; Akita, Tochigi, and Tokyo. Few patients reported to the 1996 survey were observed as fatal cases in the current study because of the high case-fatality rate during a short period. Thus, although the epidemiologic observation like this study could not provide the reason, the incidence rate might be high in these prefectures truly, not by chance. Although there was possibility that physicians in there areas, where the incidence rate of cerebrovascular disease is high, focus on the neurological findings of patients and the morbidity and mortality were seen to be high, the probability of this phenomenon seem low because of the high social and medical recognition of CJD in a recent decade by two social issues about this disease in Japan; iatrogenic CJD through dura mater graft,^2^ and bovine spongiform encephalopathy. Further studies are required to search the reason. On the other hand, Tokyo, which showed a statistically high incidence rate in the survey of 1996, did not have elevated mortality rate from CJD in the current study. The previous results might be by chance.

Yamanashi, having significantly high mortality rate, is well-known by the fact that several familial cases are clustered in the prefecture.^11^^,^^19^^-^^21^ In the current study, whether the fatal cases are suffered from sporadic CJD, familial CJD, or other types of CJD such as iatrogenic CJD, is not known. However, it is reasonable that CJD is more prevalent in Yamanashi Prefecture than in other places in Japan because there are sporadic CJD cases with the same incidence rate of the disease around Japan, plus such familial cases in Yamanashi Prefecture. On the other hand, Fukuoka Prefecture, where three familial cases were reported,^22^ did not show high mortality rate in the current study.

The other type of the CJD is iatrogenic,^15^ especially cadaveric dura mater transplantation in Japan.^2^ Not only in major neurological surgery, such as for cerebrovascular diseases and brain tumor, but also in Jannetta procedure, the dura mater were used in this country. In addition, all the surgeons did not use the mater in Jannetta procedure; the hospitals where the operation with the mater were limited. For example, 21 cases of CJD with a history of dura transplantation through the Jannetta procedure has been recognized by the surveillance system of the Japanese government, and 8 cases had the operation in one hospital (unpublished data). Several surgeons liked this technique so that the clustering of iatrogenic CJD deaths may be reasonable. Unfortunately, we have no data about the clustering of the deaths due to iatrogenic CJD because it is not classified on the vital statistics.

This study includes some limitations. First, type-specific mortality rates, such as sporadic CJD, familiar CJD, and iatrogenic CJD, were not observed because the vital statistics, which were used in this study, do not provide these data. We cannot deny the hypothesis that patients with familial CJD or CJD with cadaveric dura mater transplantation cluster in some districts. In addition, our observation included prion diseases other than CJD, such as Gerstmann-Sträussler-Scheinker disease and fetal familiar insomnia, both of which are human prion diseases, because they are classifies as A81.8 in the ICD-10th. However, "Creutzfeldt-Jakob disease" as a generic term includes Gerstmann-Sträussler-Scheinker disease and fetal familiar insomnia in Japan as well as other countries. The second limitation is data qualification. The diagnosis of CJD is due to death certificates, and no information supporting the certainty of the diagnosis cannot be obtained from the vital statistics. The third limitation is the lack of information about genotype of the disease because of the limitation of the data. Even sex-specific SMRs were not observed because the vital statistic data were provided as uniform figures including both sexes. Finally, we used the census data in 2000 as denominators of the age- and sex-specific mortality rates for the standard population, but the problem is not so serious because the census year was one of the calendar years observed in this study. In spite of these limitations, however, we consider this study is still meaningful because this is the first observation of the heterogeneous distribution to consider mortality from CJD in Japan.

In conclusion, some prefectures with high mortality rate from CJD exist in Japan. Some of them had high incidence rate in a survey conducted in 1996 as well.
